# A screen of FDA-approved drugs identifies inhibitors of protein tyrosine phosphatase 4A3 (PTP4A3 or PRL-3)

**DOI:** 10.1038/s41598-021-89668-5

**Published:** 2021-05-13

**Authors:** Dylan R. Rivas, Mark Vincent C. Dela Cerna, Caroline N. Smith, Shilpa Sampathi, Blaine G. Patty, Donghan Lee, Jessica S. Blackburn

**Affiliations:** 1 Department of Molecular and Cellular Biochemistry, University of Kentucky, Lexington, KY 40536 USA; 2 Department of Biochemistry and Molecular Genetics, University of Louisville, Louisville, KY 40202 USA; 3 Department of Medicine, James Graham Brown Cancer Center, Louisville, KY 40202 USA; 4 Markey Cancer Center, Lexington, KY 40536 USA

**Keywords:** Drug screening, Cancer, Metastasis, Oncogenes, Cancer

## Abstract

Protein tyrosine phosphatase 4A3 (PTP4A3 or PRL-3) is highly expressed in a variety of cancers, where it promotes tumor cell migration and metastasis leading to poor prognosis. Despite its clinical significance, small molecule inhibitors of PRL-3 are lacking. Here, we screened 1443 FDA-approved drugs for their ability to inhibit the activity of the PRL phosphatase family. We identified five specific inhibitors for PRL-3 as well as one selective inhibitor of PRL-2. Additionally, we found nine drugs that broadly and significantly suppressed PRL activity. Two of these broad-spectrum PRL inhibitors, Salirasib and Candesartan, blocked PRL-3-induced migration in human embryonic kidney cells with no impact on cell viability. Both drugs prevented migration of human colorectal cancer cells in a PRL-3 dependent manner and were selective towards PRLs over other phosphatases. In silico modeling revealed that Salirasib binds a putative allosteric site near the WPD loop of PRL-3, while Candesartan binds a potentially novel targetable site adjacent to the CX_5_R motif. Inhibitor binding at either of these sites is predicted to trap PRL-3 in a closed conformation, preventing substrate binding and inhibiting function.

## Introduction

Phosphatases work in concert with kinases to control phosphorylation of proteins, lipids, and other macromolecules to regulate many cellular processes. Consequently, dysregulated phosphorylation is a hallmark of cancer and multiple other diseases. Kinases catalyze phosphorylation events and have been drug targets for decades in cancer research. The critical roles of phosphatases in oncogenesis and cancer progression are just now beginning to be appreciated, and interest has grown in developing phosphatase inhibitors. More than thirty potential oncogenic phosphatases have now been identified, with roles in cellular proliferation, differentiation, migration, and angiogenesis, among others^1^. Protein tyrosine phosphatases (PTPs), in particular, have emerged as central regulators of cancer development and progression, with their increased activity correlated to enhanced tumor formation in mouse models and worse prognosis in patients^2–5^.

Members of the phosphatase of regenerating liver (PRL) family, also known as the Protein Tyrosine Phosphatase 4A (PTP4A) family, are dual specificity phosphatases that can act on both tyrosine and serine/threonine residues^6^. While their physiologic cellular functions are largely unknown, the PRL family has been repeatedly shown to be involved in cancer progression. In particular, PRL-3 is a well-defined biomarker of metastasis in multiple cancer types, including melanoma, colorectal, and ovarian cancer, where PRL-3 expression is significantly higher in metastatic lesions compared to the primary tumor site^7–15^. In a comprehensive study of 151 patient samples across eleven common human tumors types, PRL-3 protein expression was upregulated in 80.6% of tumor samples compared to matched normal tissue^16^. High PRL-3 expression has also been associated with worse prognosis in human leukemia, breast, gastric, ovarian, and colorectal cancers^9,17–20^. Additionally, the cellular function of PRL-3 in cancer progression is now well-documented experimentally. PRL-3 overexpression in tumors results in inhibition of apoptosis, promotion of epithelial to mesenchymal transition (EMT), and enhanced migration. Additionally, PRL-3 overexpression in mouse models resulted in accelerated tumor formation and increased metastasis across a variety of tumor types^21–24^ . Conversely, PRL-3 loss has been shown to prevent tumor growth and metastasis in several in vivo models^25–27^. In one example, PRL-3 loss resulted in 50% less tumor formation in a colitis-associated colorectal cancer model^28^.

The other PRL family members, PRL-1 and PRL-2, share a high degree of sequence homology to PRL-3 and may possess similar functions. Like PRL-3, both PRL-1 and PRL-2 prevent contact-mediated growth inhibition, increase tumor growth, and enhance cell migration and invasion^29–33^. Additionally, high PRL-1 and PRL-2 expression has been reported in a variety of cancer types including cervical, hepatic, and breast cancers^34–36^. Although less studied than PRL-3, data indicate that PRL-1 and PRL-2 overexpression increases metastasis in mouse models, while their loss decreases tumor cell migration and invasion. Together these results demonstrate the importance of the PRL family both in tumor formation and cancer progression, which has made them attractive therapeutic targets.

Currently, clinically available PRL inhibitors are lacking. This is in large part due to several significant challenges associated with small molecule inhibition of the PRL family, including the high level of homology between the PRLs, which makes targeting individual PRLs difficult, and conservation of the active site among PRLs and other tumor suppressive tyrosine phosphatases, such as PTEN^37,38^. Additionally, the PRL active site is shallower, wider, and more hydrophobic than other phosphatases, making design of PRL-specific inhibitors more challenging. In spite of these obstacles, several groups have identified or developed PRL-specific inhibitors including Thienopyridone, JMS-053, Compound 43, and Analog 3^39–43^. While these compounds exhibit anti-cancer effects in vitro and in mouse xenograft studies, difficulties in formulation have thus far prevented further development into clinical and therapeutic agents. Promisingly, a humanized PRL-3 antibody was recently developed that prevents growth of PRL-3 expressing tumors, while not targeting PRL-3-negative tissues. This antibody is currently in phase II clinical trials for patients with advanced solid tumors that have failed standard therapy outcomes have yet to be measured, and this drug will take some time to be widely available if it is proven safe and effective^16,44^.

To address the immediate need for PRL-3 inhibitors in the clinic, we screened a library of 1443 FDA approved drugs for their ability to modulate the phosphatase activity of PRLs. We found one selective inhibitor of PRL-2, five selective inhibitors of PRL-3, and nine potent inhibitors of the PRL family. Two drugs from the latter group, Salirasib and Candesartan, prevented PRL-3 mediated cell migration in PRL-3 overexpressing human embryonic kidney (HEK) cells without impacting cell viability. Furthermore, these compounds were able to inhibit migration in a colorectal cancer cell line that expressed endogenously high levels of PRL-3. In silico docking of the drugs to PRL-3 showed that Salirasib binds to PRL-3 in the same site proposed for the research-grade PRL-3 inhibitor, JMS-053, while Candesartan binds a secondary site in PRL-3 that has not previously been targeted. These drugs appear to function allosterically by locking PRLs in the closed confirmation. Together, our results indicate that it may be possible to repurpose FDA-approved drugs to block PRL-3 activity in human cancer cells. These drugs may also provide some insight into the structures of compounds that are best able to selectively target PRL-3 and can be used as chemical probes to uncover biological functions of PRL-3.

## Results

### Identification of FDA-approved drugs that inhibit PRL activity

**Figure 1:**
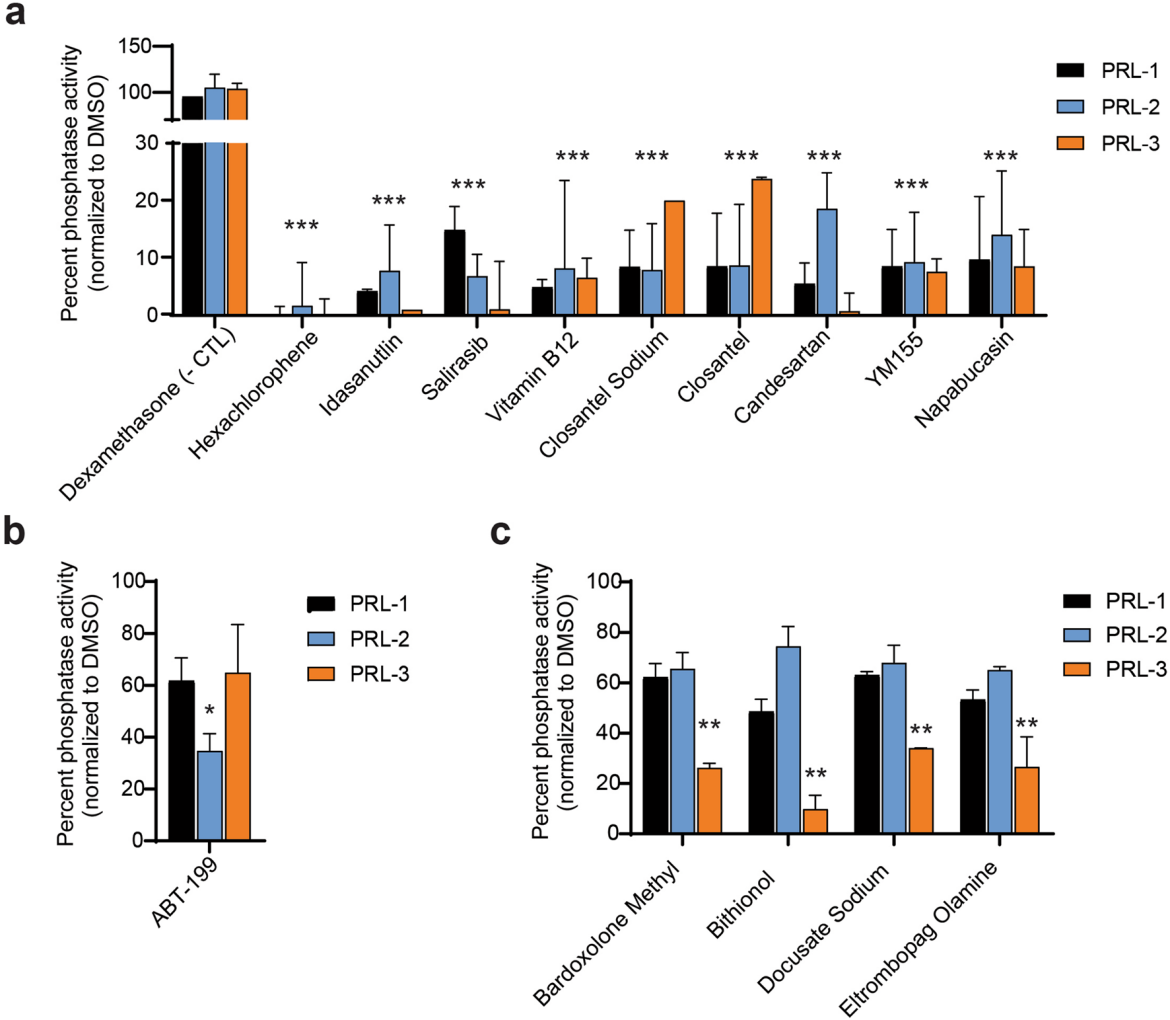
Multiple FDA-approved compounds inhibit the phosphatase activity of PRL proteins. (**a**) Validation of drugs that were positive hits in the initial FDA-drug screen and significantly reduced PRL phosphatase activity across multiple tests. The FDA-approved drug Dexamethasone, which has no effect on PRL-3 phosphatase activity, was used as a negative control (- CTL). (**b**) ABT-199 demonstrated selectivity in inhibiting PRL-2. (**c**) Validation of PRL-3 specific inhibitors. All assays were run at a final PRL protein concentration of 2.5 µM, a drug concentration of 40 µM, and DiFMUP concentration at the previously reported K_M_ of the protein. Bars represent the average phosphatase activity of the initial screen plus two additional independent experiments, in triplicate. Error bars represent standard deviation. *p < 0.05, **p < 0.001, ***p < 0.0001 by either two-way ANOVA with Dunnet’s correction (**a**) or one-way ANOVA with Tukey’s HSD (**b**, **c**).

Phosphatase activities of recombinantly expressed PRL-1, PRL-2, and PRL-3 were evaluated based on their ability to hydrolyze the synthetic substrate, phospho-tyrosine analog 6,8-Difluoro-4-methylumbelliferyl phosphate (DiFMUP). DiFMUP hydrolysis could be significantly inhibited by three research-grade PRL inhibitors: the rhodamine-derivative PRL Inhibitor I^45^ , Analog 3^41^ , and Thienopyridone^40^ (Supplemental Fig. 1). Similar to prior reports, Thienopyridone was most effective, blocking PRL activity by ~ 95%, and was used as a positive control for PRL inhibition throughout the rest of our studies. We next screened 1,433 FDA-approved drugs for their ability to inhibit PRL dephosphorylation of DiFMUP. Phosphatase activity of the PRL after drug treatment ranged from 0 to 150% of the dimethylsulfoxide (DMSO) control. Fifty-three compounds that emitted fluorescence autonomously were excluded due to the potential interference with data analysis. The mean phosphatase activity across the remaining 1,380 drugs was calculated for each PRL, and a compound was considered a hit in this assay when the drug reduced the phosphatase activity of the PRL to three standard deviations lower than the mean phosphatase activity (Supplemental Table 1). The DiFMUP assay was then repeated on the initial hits, which confirmed nine broad inhibitors that blocked the phosphatase activity of PRL-1, PRL-2, and PRL-3 by more than 80% (Fig. 1a, Supplemental Fig. 2). Additionally, there was a single drug that preferentially inhibited PRL-2, and four drugs that preferentially inhibited PRL-3 over other PRLs (Fig. 1b, c).

**Figure 2:**
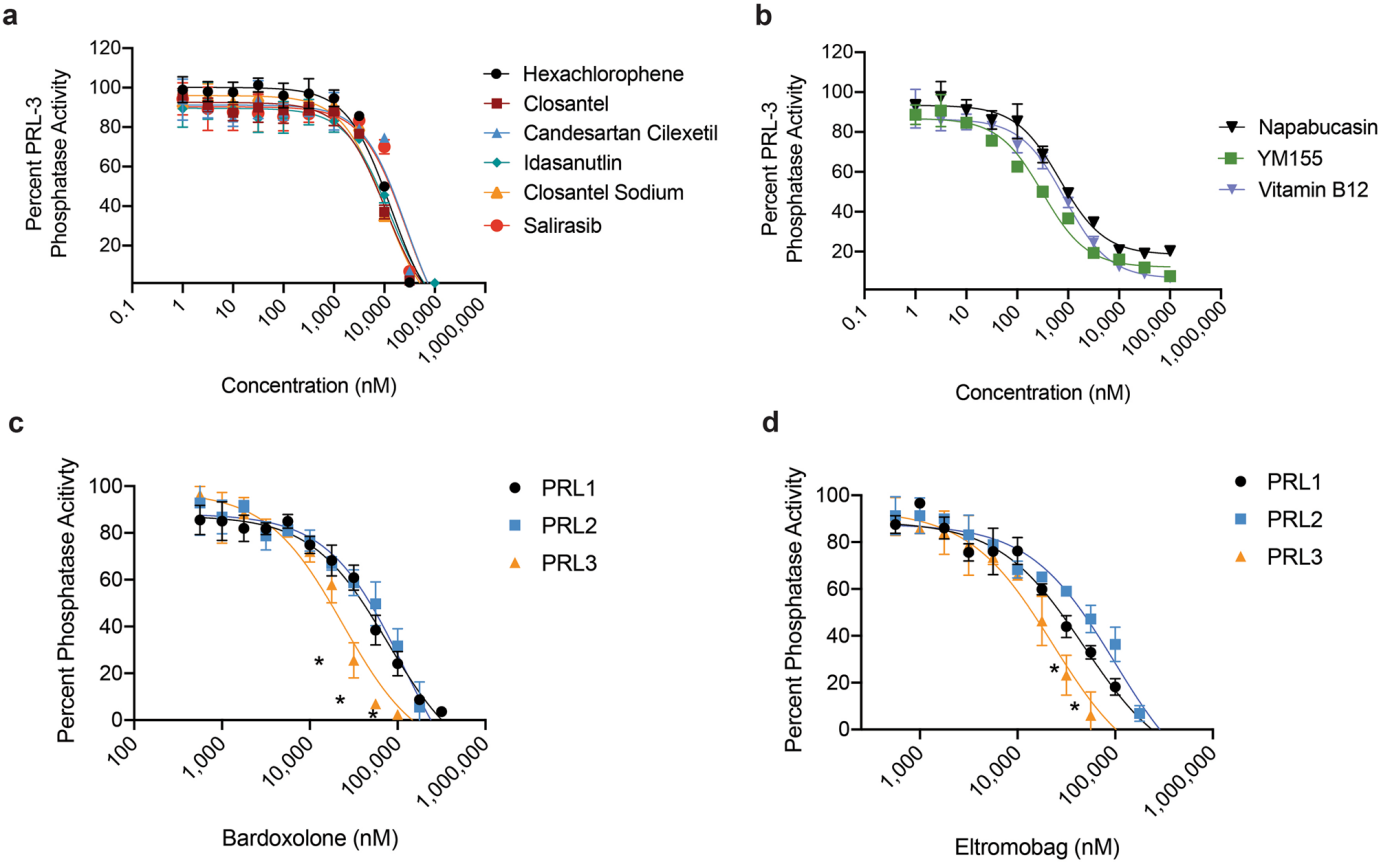
Dose dependent inhibition of PRL family members with FDA-approved drugs. Dose response curves showing DiFMUP phosphatase activity of PRL-3 when treated with doses ranging between 1 nM and 100 µM of each drug. Inhibition of PRL-3 phosphatase activity by the broad PRL family inhibitors are shown, and drugs are subdivided into those with (**a**) high IC_50_ values and (**b**) low IC_50_ values. A subset of compounds preferentially inhibited PRL-3, including (**c**) Bardoxolone and (**d**) Eltromobag. All assays utilized a final protein concentration of 2.5 µM with DiFMUP concentration at the K_M_ of the protein, and were run in technical duplicates in three independent experiments. Data represent mean phosphatase activity and error bars represent standard deviation between assays. * *p* < 0.01 comparing PRL-3 phosphatase activity to that of PRL-1 and PRL-2 at the indicated drug dose, using a two-way ANOVA with Dunnet’s correction.

**Table 1:** Calculated IC_50_ values in μM from broad and specific inhibitors of PRL phosphatase activity.

	PRL-1	PRL-2	PRL-3
**Broad PRL inhibitors**			
Candesartan	69 ± 8	80 ± 15	28 ± 4
Closantel	17 ± 3	14 ± 5	11 ± 0.5
Closantel Sodium	22 ± 8	18 ± 1	10 ± 0.4
Hexachlorophene	31 ± 4	35 ± 10	13 ± 1
Idasanutlin	20 ± 3	16 ± 2	13 ± 0.4
Napabucasin	0.4 ± 0.2	1.1 ± 0.3	0.7 ± 0.1
Salirasib	53 ± 5	118 ± 22	27 ± 3
Vitamin B12	0.7 ± 0.2	0.9 ± 0.2	0.9 ± 0.1
YM155	0.3 ± 0.1	0.7 ± 0.1	0.3 ± 0.1
**PRL-3 specific inhibitors**			
Bardoxolone	85 ± 43	136 ± 75	24 ± 5*
Bithionol	79 ± 25	115 ± 34	33 ± 2*
Docusate Sodium	94 ± 26	140 ± 44	43 ± 5*
Eltromobag	56 ± 10	105 ± 19	26 ± 11*
Eltromobag Olamine	61 ± 15	166 ± 22	27 ± 4*
Embelin	114 ± 16	124 ± 60	52 ± 9*

*p < 0.05 between IC50 values for PRL-3 and PRL-1 and PRL-2 by one-way ANOVA with Tukey’s HSD.

All broad inhibitors blocked PRL phosphatase activity in a dose dependent manner, between 1 nM and 100 µM. The broad PRL inhibitors loosely separated into those with high IC_50_ values (greater than 1 µM), and low IC_50_ values (less than 1 µM) for PRL-1, PRL-2 and PRL-3 (Fig. 2a, b, Supplemental Fig. 3). Additionally, the PRL-3 specific inhibitors were also able to selectively inhibit PRL-3 phosphatase activity at a significantly lower IC_50_ than PRL-1 and PRL-2 (*p* < 0.0001), although they did inhibit these phosphatases to an extent (Fig. 2c, d, Supplemental Fig. 4). On average the broad PRL inhibitors had an IC_50_ value threefold lower than the specific PRL-3 inhibitors (Table 1), so we focused on the broad PRL inhibitors. The specific PRL-2 inhibitor, ABT-199, is also a potent inhibitor of Bcl-2^46^. Due to the probable associated toxicity at the dose of drug needed for effective PRL-2 inhibition, this drug was not pursued further.

**Figure 3:**
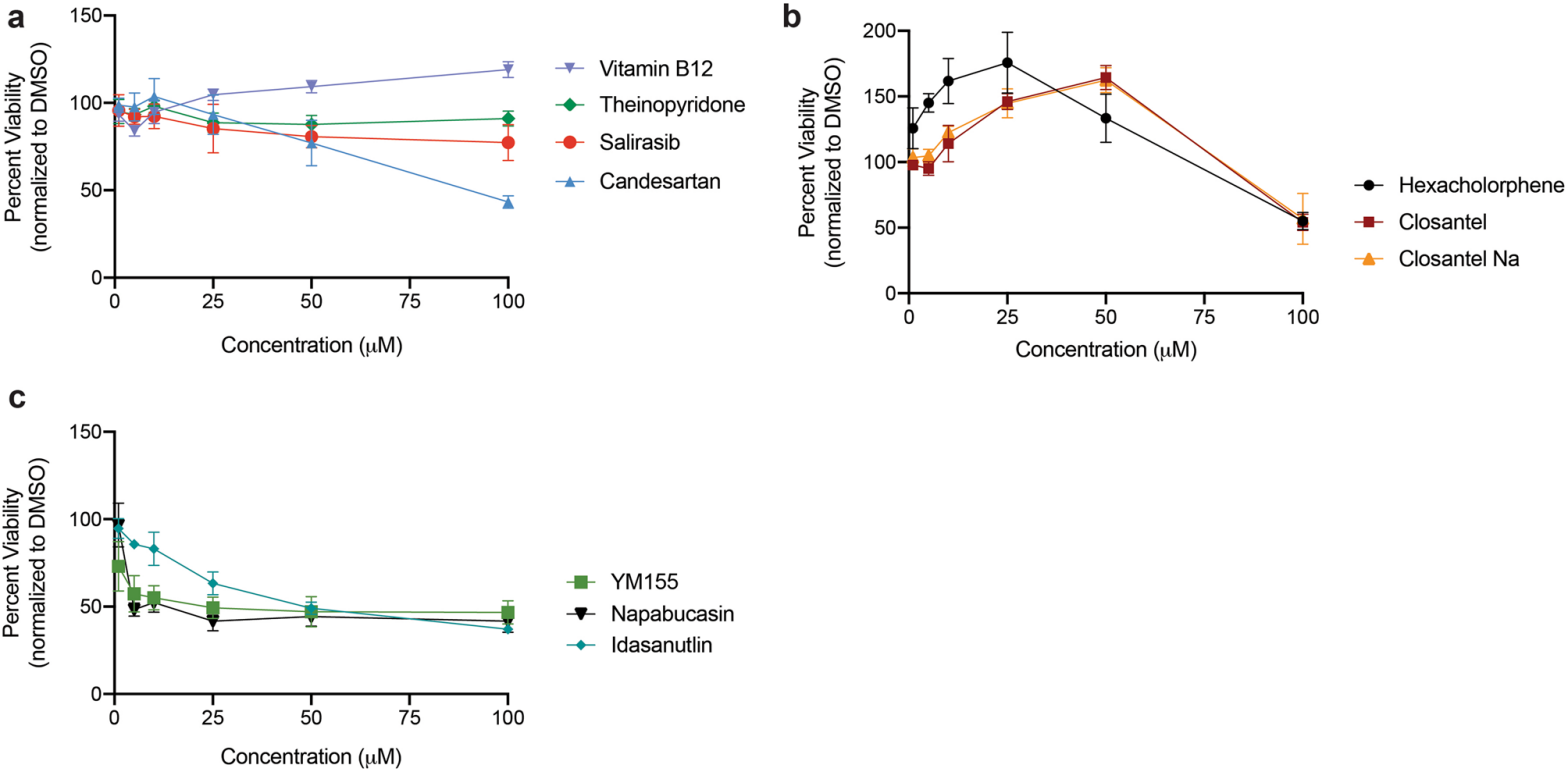
Salirasib and Candesartan are non-toxic PRL inhibitors. HEK293T cells were cultured with drugs at a range of concentrations and viability was assessed after 16 h. Drugs were sub-divided into those that were (**a**) minimally toxic, (**b**) caused increased viability, and (**c**) were highly toxic. Viability was measured as cellular reduction potential using MTT dye, and normalized to 1% DMSO control. Assays were run in triplicate in three independent experiments. Data represent mean viability and error bars represent standard deviation between assays.

**Figure 4:**
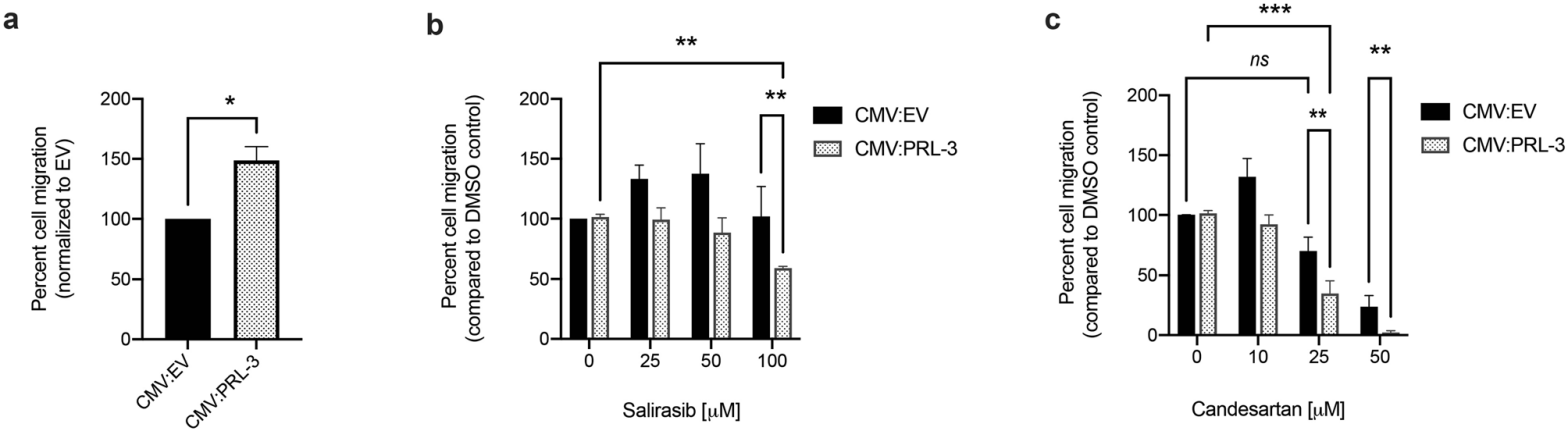
Salirasib and Candesartan inhibit PRL-3 induced cell migration in HEK293T cells. (**a**) Quantification of cell migration of HEK293T cells transfected with a either PRL-3 expression vector or empty vector control. Cells from (a) were treated with either (**b**) Salirasib or (**c**) Candesartan at the doses indicated. The zero drug dose was a 1% DMSO control, and each data set was compared to the DMSO control group within that data set. Both drugs inhibited cell migration to a greater extent in the PRL-3 overexpressing cells, compared to empty vector (EV) control. *p = 0.089 using one tailed student t test with Holm Sidak correction, **p = 0.03, ***p = 0.001, *ns* = no significant difference, using a two-way ANOVA with Dunnett’s correction.

### A subset of compounds is non-toxic in HEK293T cells at doses that are inhibitory to PRL-3

Drugs were next tested for off-target effects on the cell viability at the IC_50_ of PRL-3 inhibition. The immortalized human embryonic kidney cell line 293T (HEK293T) expresses very low levels of PRL-3 and any changes in viability with drug treatment would be due to off-target toxcity. Cells were treated with doses of drug and analyzed by an MTT (3-(4-5-dimethylthiazol-2-yl)-2,5-diphenyltetrazolium bromide) assay, which measures cellular metabolic activity as a is a read-out of the cell viability. Salirasib, Vitamin B12 and the positive control for PRL-3 inhibition, Thienopyridone, had no effect on the viability at drug doses ranging from 1 to 100 µM (Fig. 3a) after 16 hours of incubation with the compounds. Candesartan showed insignificant effect on viability up to the concentration of 50 µM but viability dropped significantly at 100 µM, indicating potential toxicity of the compound at concentrations well above the IC_50_ for PRL-3 inhibition. Hexachlorophene and Closantel increased MTT absorbance in 293T cells (Fig. 3b). Finally, Idasanutlin, YM155, and Napabucasin decreased viability of 293T cells (Fig. 3c), most likely due to these drugs having a known higher affinity for proteins involved in apoptotic pathways^47,48^ and potentially other off-target effects. As the ultimate goal of this study is to identify drugs that could be used as anti-cancer therapy in PRL-3 expressing tumors, we eliminated drugs that enhanced cell viability, and focused on those that decreased or caused no change in cell viability.

### FDA-approved drugs blocked PRL-3 induced cell migration in human cancer cells

PRL-3 is well-established to promote cell migration. Scratch assays were used to test whether broad PRL inhibitors would alter the migratory phenotype of PRL-3 overexpressing HEK293T cells and human colorectal cancer cells that have endogenously high levels of PRL-3. Transfection of a PRL-3 expression vector increased PRL-3 expression in HEK293T cells by fourfold and enhanced cell migration by 40% (*p* = 0.02, Fig. 4a, Supplemental Fig. 5). Empty vector and PRL-3 transfected cells were then treated with the broad PRL inhibitors at concentrations that were previously found to not affect cell viability by more than 25%. Treatment with two of the drugs, Salirasib and Candesartan, resulted in a significant inhibition of cellular migration in PRL-3 overexpressing cells, compared to DMSO treatment (Salirasib, *p* = 0.03 and Candesartan *p* = 0.001, Fig. 4b, c) with no corresponding reduction in migration in control cells. These data indicate that these drugs can directly inhibit PRL-3 to impact cellular migration. Other drugs, including Idasanutlin, YM155, and Napabucasin had less significant effects on the migration of PRL-3 expressing cells, compared to control cells, or impacted migration in both PRL-3 overexpressing and control cells equally, and were therefore excluded from further analysis (Supplemental Fig. 6). Neither Salirasib nor Candesartan had a negative effect on the activity of 18 other phosphatases (Supplemental Table 2).

**Figure 5:**
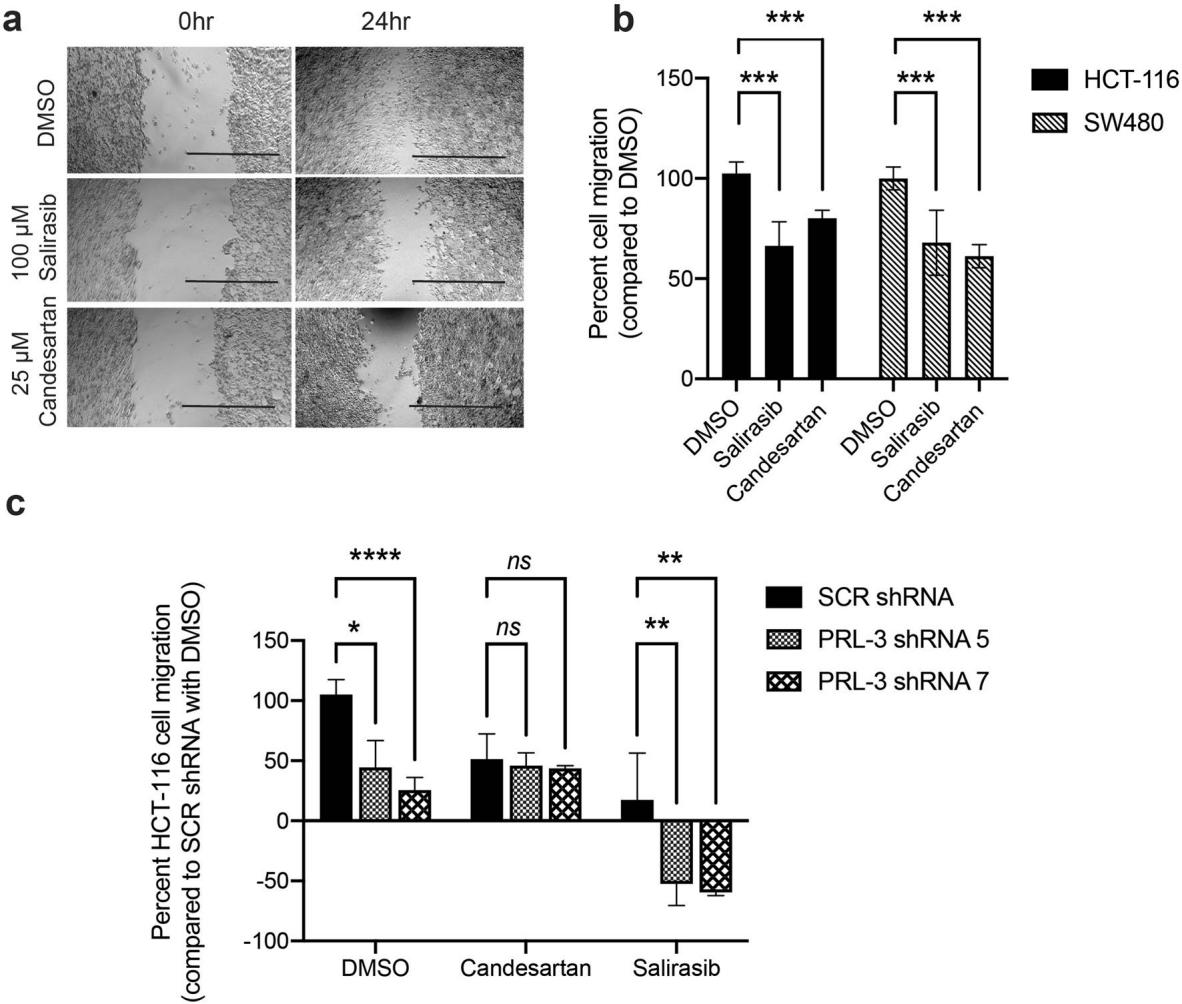
Salirasib and Cadnesartan inhibit PRL-3 mediated cell migration in human colorectal cancer cells. (**a**) Representative images of migration of the human colorectal cancer cell line HCT116 when treated with 1% DMSO, 100 µM Salirasib or 25 µM Candesartan. (**b**) Quantification (**a**) across three independent experiments, in triplicate, in both HCT116 and SW480 cells. Migration was measured by quantifying the empty area in the image of the entire well at 0 and 24 h post-scratch using ImageJ and calculating the difference. Percent migration was measured as the area migrated 24 h after scratch normalized to the area migrated by DMSO control. (**c**) Migration of HCT116 cells that were transfected with either scrambled or PRL-3 shRNA and then treated with 1% DMSO, 100 µM Salirasib or 25 µM Candesartan. Data were quantified as above, except that all data sets were normalized to the Scrambled shRNA transfected cells treated with DMSO. Negative values indicate that the empty area was larger at 24 h than the 0 timepoint. All assays were run in duplicate wells, in 3 independent experiments. Bars indicate the mean percent cell migration, and error bars represent standard deviation between assays. ***p = 0.026 using one-way ANOVA with Tukey’s HSD, *p = 0.039, ****p = 0.0021, **p = 0.02, *ns* = not significant, analyzed by a two-way ANOVA with Dunnett’s correction.

**Figure 6:**
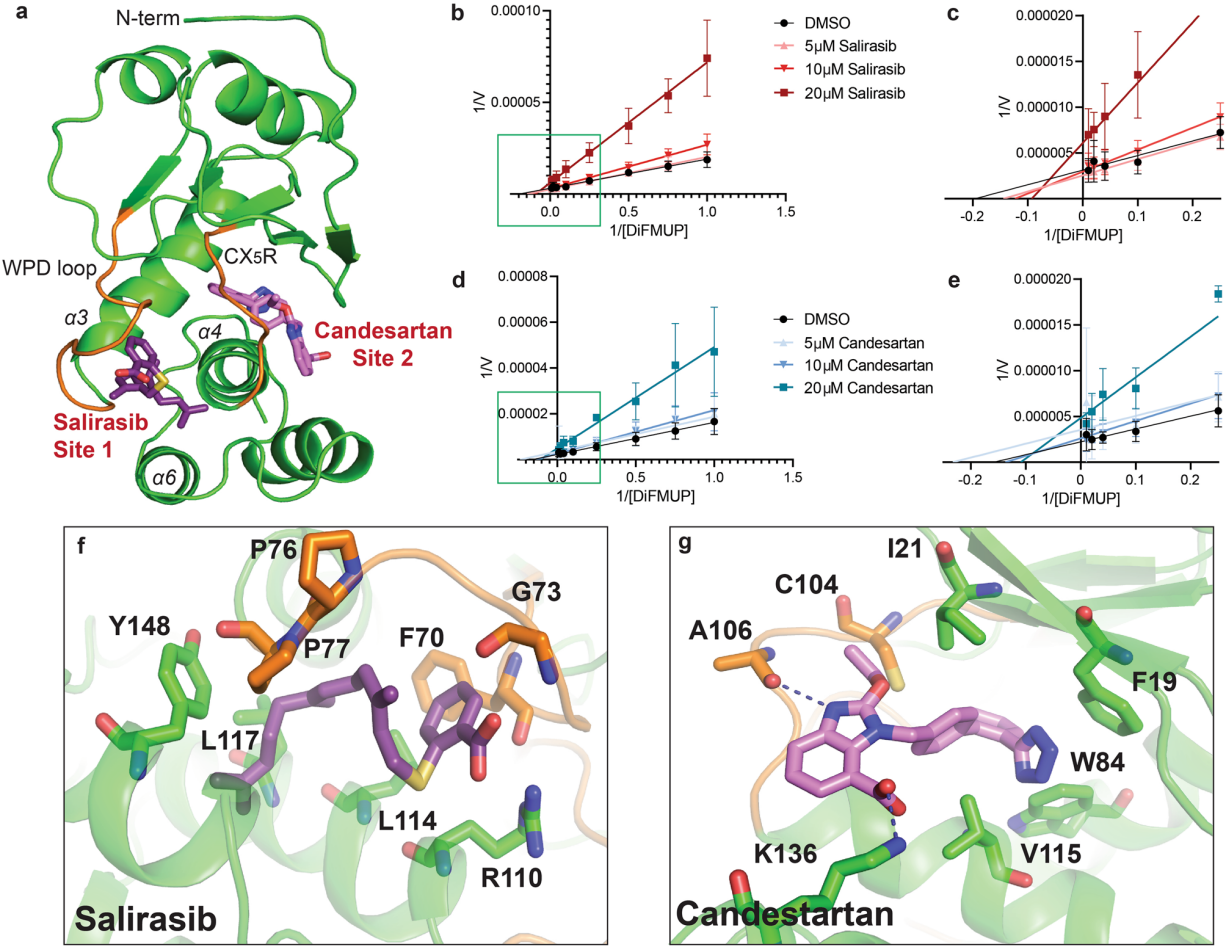
Molecular docking experiments reveal that top hits from screening FDA-approved drugs bind to allosteric sites. (**a**) Representive compounds, Salirasib and Candesartan, are shown bound to two identified allosteric sites adjacent to the active site looops (orange) in the closed conformation of PRL-3 (PDB: 2MBC). Lineweaver–burk plots indicate that Salirasib (**b**, **c**) and Candesartan (**d**, **e**) are non-competitive inhibitors to the substrate, supporting the docking results. Graphs in (**c**) and (**e**) are insets of the boxed areas in (**b**) and (**d**). Assays were run with technical duplicates and performed in 3 independent replicates. Error bars represent standard deviation between assays. Proposed binding modes of Salirasib (**f**) and Candesartan (**g**) on PRL-3, with residues in close proximity to the bound molecule identified.

Next, we tested whether Salirasib and Candesartan could decrease cellular migration in human colorectal cancer cells that express high levels of endogenous PRL-3^12,22^. We found that both drugs significantly inhibited the migration of the HCT116 and SW480 cells by > 30% (*p* = 0.03, Fig. 5a, b). This drug-induced change in migration was not due to any significant change in proliferation or apoptosis rates in the cells at 24–48 h post-treatment (Supplemental Fig. 7). Although direct PRL-3 substrates are largely unknown, the ERK signaling pathway has previously been shown to be affected by PRL-3 activity in a variety of cancer cells, where it plays a role in cell migration and invasion^49,50^. Both Salirasib and Candesartan decreased phospho-ERK levels in HCT116 cells to a similar extent as the research grade PRL-3 inhibitor, Thienopyridone (Supplemental Fig. 8).

We next transfected HCT116 cells with shRNA that we have previously found to be effective against PRL-3^51^, producing a > 50% reduction in PRL-3 protein (Supplemental Fig. 5), and saw the expected decrease in colorectal cancer cell migration (Fig. 5c, Supplemental Fig. 9). Candesartan treatment of cells with PRL-3 knock-down showed no additional impact on migration, suggesting that this drug largely acts through PRL-3 to reduce cell migration (Fig. 5c). Interestingly, colorectal cancer cells treated with Salirasib in combination with PRL-3 shRNA had significantly reduced migration compared to control shRNA, with the scratch widening after 24 h, indicating some die-off of PRL-3 shRNA/Salirasib treated cells that is not present in the control shRNA/Salirasib treated cells (Supplemental Fig. 9). These data indicate that Salirasib is not as specific for PRL-3 as Candesartan. As a known Ras inhibitor, Salirasib may target other important pathways in colorectal cancer cells that can synergize with PRL-3 knockdown to impact both cell viability and migration.

### In silico docking of Salirasib and Candesartan on PRL-3

Due to the lack of currently available structure of monomeric PRLs in complex with any inhibitor, inhibitor binding sites are largely unknown. Attempts by other groups to obtain co-crystals of PRL-3 with inhibitors have thus far been unsuccessful. Molecular docking was therefore employed to identify the potential binding sites of the drugs used in our assay, including the potent inhibitors Salirasib and Candesartan, and to further understand their mechanisms of action. The docking simulations show that, expect for Hexachlorophene, all of the drugs bind preferentially (with lower binding energies) to the closed conformation of PRL-3 (Supplemental Table 3, Supplemental Fig. 10). In the closed form, two major binding sites were identified: Site 1, which is flanked by the WPD loop and helices α3, α4, and α6 and has been previously predicted to be an allosteric binding site for the research-grade PRL-3 inhibitor, JMS-053^11^, and Site 2, a potential secondary allosteric site adjacent to the PRL-3 CX_5_R motif and bound by helix α4 and the β-sheets (Fig. 6a). Lineweaver-Burke analyses show that both Candesartan and Salirasib reduce apparent V_max_ but do not affect the K_M_ and are non-competitive inhibitors to the DiFUMP substrate (Fig. 6b–e). Therefore, both inhibitors bind sites other than the active site, consistent with our in silico models indicating that both compounds bind to potential allosteric sites.

In silico modeling suggests that Salirasib binds in the allosteric Site 1 with a free energy of binding of − 10.46 kcal/mol. This pocket is lined with hydrophobic residues that likely stabilizes the farnesyl tail of the drug. The salicylic acid group of Salirasib is then free to form hydrogen bonding, in this case with the backbone amide and carbonyl groups of G73 (Fig. 6f), likely stabilizing PRL-3 in a closed conformation. Candesartan binds at the potentially new allosteric Site 2 with an estimated free energy of binding of − 10.26 kcal/mol for the closed site. In particular, it fits in a shallow binding pocket lined with hydrophobic residues, similar to Site 1, and can form electrostatic interactions with PRL-3 through the sidechain of K136 and the backbone carbonyl of A106 (Fig. 6g). The low docking scores are expected due to the low molecular weight of the drugs. The drugs and the sites are also largely hydrophobic. Regardless, these docking simulations show a potential mechanism for PRL-3 inhibition by Salirasib and Candesartan. Additionally, Site 2 offers an additional targetable site that to our knowledge has not been pursued specifically in the past.

As a validation of these results, drugs that were used as controls in experimental work were docked. Dexamethasone was used as a negative control, while the rhodanine derivative PRL-3 Inhibitor was used as a positive control in the drug screen. In both conformations, dexamethasone was the lowest ranked, while PRL-3 Inhibitor is among the higher ranked. Finally, a farnesyl tail was docked to verify binding of the farnesyl derivative Salirasib. Blind docking identified the same binding pocket in Site 1 (Supplemental Fig. 11).

## Discussion

PRL-3 is highly expressed in many cancer types and is a proven oncoprotein. It has well-established roles in tumor cell invasion and metastasis, and data suggests it may also be involved in cancer cell proliferation and drug resistance^52,53^. There have been increasing efforts to identify PRL-3 inhibitors. A screen of the Korea Chemical Bank identified the rhodanine derivatives CG-707 and BR-1 as PRL inhibitors, but further analysis showed these to be fairly nonspecific^45^. Screening of the Roche chemical library identified Thienopyridone as a PRL-3 inhibitor that reduced tumor growth by interfering with cell adhesion, although it is too toxic for in vivo use^40^. Further analysis of Thienoypridone, relying on active site mimicry and in silico structural modeling, led to the discovery of an additional PRL-3 inhibitor, Analog 3^41^. A subsequent structural activity relationship study identified iminothienopyridone 13 (JMS-053), an analog with lower toxicity and increased potency than Thienopryidone^42^. This compound is notable because it is the first PRL-3 small molecule inhibitor used in vivo, where it inhibited cancerous cellular growth in a multi-drug resistant ovarian xenograft model. However, the solubility of JMS-053 is poor, and it requires further study to determine pharmacokinetics, toxicity, and efficacy in humans. Finally, a specific PRL-3 binding antibody has been developed, which is currently the only inhibitor capable of selectively targeting PRL-3 over other family members and is capable of preventing tumor growth using in vivo model of both hepatic and gastric cancers^16,44^. Although antibody treatments are an effective strategy for targeting proteins in a variety of diseases, the associated costs are significantly higher than using a small molecule inhibitor, and this antibody will also require clinical trials for safety and efficacy. Although efforts are underway to improve each of these compounds and biologics, it can take many years to bring a drug from development to the clinic.

Through our screen of > 1400 FDA-approved drugs, we identified one compound, Candesartan, that significantly reduced PRL phosphatase activity. Candesartan is FDA-approved as an angiotensin receptor blocker to treat high-blood pressure, and has not previously been used as an anti-cancer therapy. Angiotensin receptors are G-protein coupled receptors that are typically involved in transmitting vasoconstrictive stimuli in in blood vessels, and are variably expressed in cancer cells. Interestingly, Candesartan has been found to interact with the proteins *Plekhj1*, *Sh2b3*, and *DotL1*, all of which are involved in various signaling pathways in cancer cells. Our data indicate that Candesartan blocks PRL-3 phosphatase activity, can revert PRL-3 induced migration in HEK293T cells, and can reduce migration of colorectal cancer cells that express high levels of PRL-3, with no impact on migration in the same cells with PRL-3 knocked down via shRNA. While these data suggest some specificity of Candesartan for PRL-3, additional studies are needed to define the other pathways and cellular responses that might be affected by this drug across different cancer types.

A second drug identified in this screen, Salirasib, is an S-farnesyl cysteine analog that functions to dislodge Ras-isoforms from their membrane anchoring sites, leading to Ras degradation. Many cancers rely on dysregulated Ras signaling for a variety of cellular processes, including cell growth and migration. While Salirasib blocked PRL-3 phosphatase activity in a cell-free system, it is difficult to ascribe the cellular functions of Salirasib strictly to its interactions with PRL-3—Salirasib treatment of colorectal cancer cells with PRL-3 knock-down appeared to die to an extent not seen with either PRL-3 shRNA or Salirasib treatment alone, suggesting multiple mechanisms of action for this drug, perhaps in synergy with PRL-3 loss. Additionally, similar to Ras, PRL-3 is farnesylated and localized to the cell membrane. PRL-3 would therefore be similarly outcompeted for membrane anchoring sites by Salirasib, suggesting that the drug may both be blocking PRL-3 activity through interaction with the protein, and causing PRL-3 mis-localization by preventing membrane binding.

The lack of available structural information of PRL-3 alone or in complex with an inhibitor makes it challenging to identify targetable residues in PRL-3 or propose binding sites for potential inhibitors. However, docking functionally validated PRL inhibitors via in silico approaches can provide useful information on PRL-3 interactions with drugs. We found that Salirasib binds to a previously identified binding pocket adjacent to the conserved WPD loop; the allosteric inhibitor JMS-053 was also proposed to bind to this site^11^. Candesartan binds to a secondary pocket at the opposite side of the active site, adjacent to the CX_5_R motif. Both of these binding sites are lined with hydrophobic residues that may allow binding of highly hydrophobic small molecules, like Candesartan (logP of 6.1) and Salirasib (logP of 6.8). To our knowledge, this secondary allosteric site has not been previously identified before as a binding site for inhibitors of PRL-3 and provides an alternative target site for in silico screens to identify novel molecules targeting PRL-3. Additionally, while Salirasib and Candesartan were able to inhibit PRL-3 phosphatase activity and affect cellular function, the IC_50_s were in the µM range. Future work modifying these compounds may be useful to increase the strength of binding and improve their potency.

Our models also show that drug binding might be sensitive to PRL-3 conformation. The WDP loops and the CX_5_R motif (or P loop) harbor the catalytic residues of classical dual-specificity phosphatases. The two allosteric sites identified are located adjacent to these loops. The catalytic cycle of PRLs and other classical phosphatases are characterized by large structural rearrangements primarily in the WPD loop and surrounding region. As PRL-3 transitions to its open state, the catalytic loops move a total of about 10 Å towards each other, moving the catalytic residues into close proximity. This includes the nucleophilic cysteine (P loop, C104 in PRL-3), the aspartic acid (WPD loop, D72) that acts as the general base/proton donor, and the conserved arginine (P loop, R110) that stabilizes the phosphoenzyme intermediate. Our modeling suggests that the drugs have a lower binding energy for the closed PRL-3 conformation—this indicates a possible mechanism of action where these small molecules trap PRL-3 in the closed conformation, preventing active site structural rearrangements, which is necessary for catalysis^54,55^.

Finally, targeting protein phosphatases such as PRL family members has been challenging due to high sequence similarity and the conserved active sites among protein tyrosine phosphatases. Additionally, small molecule inhibitors have little selectivity between the PRLs, since the PRL active site is wide and shallow; the most effective PRL-3 inhibitors identified in this study also targeted the entire PRL family. Allosteric inhibition of PRLs will provide an alternate strategy for the identification and development of more specific PRL-3 inhibitors. Thus far, two potential allosteric sites are identified for PRL-3 and further studies are needed to experimentally validate both sites, as well as the possibility of targeting them with small molecules. Several drugs, including Bardoxolone and Eltromobag, showed greater specificity for PRL-3 over PRL-1 and PRL-2. The properties of these drugs may be useful in informing future development of novel PRL-3 inhibitors.

## Methods

### Recombinant protein expression

PRL full length protein sequences fused to an N-terminal 6xHis-tag was cloned into pET28b bacterial expression vector. Proteins were expressed in One Shot BL21 Star bacteria (Invitrogen, Cat # C601003) by induction with 0.5 mM IPTG (Fisher Scientific, Cat # BP175510) for 16 h. Cells were resuspended in 10 ml of lysis buffer [300 mM NaCl (VWR Cat. No. BDH9286), 20 mM Tris pH 7.5, 10 mM Imidazole pH 8.0 (Sigma-Aldrich I2399), 1:1000 protease inhibitor cocktail (Sigma-Aldrich Cat. No. P8465)] per gram of cell pellet and lysed using a microfluidizer (Avestin, EmulsiFlex-C5). Protein was isolated using Ni–NTA Resin (VWR, Cat # 786–940) and eluted with 2 mL of elution buffer (300 mM NaCl, 20 mM Tris pH 7.5, and 250 mM Imidazole pH 8.0). Following cleavage with TEV protease, samples were reapplied to Ni–NTA column to remove uncleaved protein as well as TEV. Samples were further purified using an S200 column on a Superdex 10/300 in buffer containing 100 mM NaCl and 200 mM HEPES pH 7.5. Purified fractions were then run on 4–20% Mini-PROTEAN TGX Stain-Free (Bio-Rad). The purest fractions were pooled, concentrated together, flash frozen and stored at -80 °C.

### Drug panel and other reagents

The library of FDA-approved drugs was from Selleck (L1300). For further testing, single drugs were purchased as listed in Supplemental Table 4. Thienopyridone was generously provided by Dr. Zhong-Yin Zhang (Purdue University). PRL-3 overexpressing construct was made by cloning full length PRL-3 cDNA into p3XFLAG-CMV-14 expression vector (Sigma, Cat # E7908).

### In vitro phosphatase assay

In 384 well plates, 2.5 μM recombinant PRL-1, PRL-2, or PRL-3 was combined with DiFUMP (Life Technologies, Cat # E12020) at the K_M_ of each protein in reaction buffer (20 mM Tris–Cl, pH 7.5, 150 mM NaCl, 10 mM DTT) as previously reported^41^. Briefly, protein was diluted in reaction buffer and allowed to incubate at 4 °C for 20 min to allow for full reduction of the active site. DiFMUP, drug, and protein were added, and plates were incubated for 20 min at room temperature. Fluorescence intensities were measured on a Cytation 5 plate reader (Biotek) at an EX:360 nm and EM:460 nm. Data were normalized to vehicle control and IC_50_ values were calculated on GraphPad Prism version 8. Inhibition selectivity for PRLs over a panel of other phosphatases was determined using PhosphataseProfiler (Eurofins, Cat # PP260).

### Cell culture

HEK293T, HCT116, and SW480 human cell lines, from ATCC, (CRL-3216, CCL-247, and CCL-228 respectively) were maintained in 1X DMEM with glutamine and glucose (Gibco, Cat # 11,965–092) supplemented with 10% FBS (Atlanta Biologicals, Cat # S11150H) and Penicillin/Streptomycin (Fisher Scientific Cat #,10,378,016). Cells were cultured at 37 °C in a humidified incubator with 5% CO_2_.

### PRL-3 overexpression and shRNA knockdown

pENTR-PRL-3 (previously described^51^) was cloned into pLKO-CMV-puro using LR clonase (Thermofisher, 11,791,019) according to manufacturer’s directions. pLKO-CMV empty vector (EV) and pLKO-CMV:PRL-3 were transfected into HEK293T using Lipofectamine 3000 (ThermoFisher, L3000015) according to manufacturer’s protocol. Transfected cells were selected with 1 µg/mL puromycin in complete media to establish the cell line, then were maintained under 0.5 µg/mL puromycin in complete media. For PRL-3 knockdown, pLKO.1-scrambled shRNA and pLKO.1-PRL-3 shRNA^51^ plasmids were transfected into HCT116 cells using Lipofectamine 3000 (Thermo Fisher, Cat # L3000015) according to the manufacturer’s directions. Cells were selected for 48 h in 1 µg/mL puromycin before use in assays.

### Scratch assay

Cells were seeded into duplicate wells of a 48 well plates at a density of 2 × 10^5^ cells per well for HEK293T or 4 × 10^5^ for HCT116 and SW480. Cells were recovered overnight and were scratched using a p20 pipette tip the following day. Compounds were added at the concentrations indicated. Images were obtained immediately after the scratch and after a 24 h incubation with the drug on an Evos FL inverted microscope (ThermoFisher). The area within the scratch was calculated using ImageJ. The area migrated was measured as the difference in wound area at 0 h and 24 h and the percent change in migration was calculated by dividing the difference in wound area of treatment by the difference in wound area of the control.

### Viability assay

Cells were seeded into duplicate wells of a 96 well plate at a density of 2 × 10^4^ cells per well and allowed to recover for 24 h. Cells were then treated with drug or DMSO at the indicated concentrations for 16 h. Following drug treatment, 10 μL of 5 mg/ml MTT (Sigma, M5655) was added to each well and cells were incubated for 4 h. Finally, media were removed, and the dye was solubilized in 100 μL of 0.1 M HCl in isopropanol. Absorbance was measured at 570 nm and 690 nm with final absorbance measurements as 570–690 nm.

### Click-IT Edu assay

The cell proliferation assay was performed using the Click-IT Edu cell proliferation kit from (Thermo Fisher, Cat # C10419) according to the manufacturer’s protocol. Briefly, HEK293T, HCT116 and SW480 cells were plated in 6 well dishes at 4 × 10^5^ cell seeding density and treated with DMSO, Candesartan and Salirasib at the indicated concentrations. Edu was added 2 h prior to collecting the cells at 24 h and 48 h time points and the assay was performed according to the instructions from the manual. Flow cytometry with AlexaFluor647 conjugated azide dye was used to determine the cellular proliferation and DAPI was used for the DNA content.

### Annexin V staining

HEK293T, HCT116, and SW480 cells were plated in 6 well dishes at 4 × 10^5^ cell seeding density and treated with DMSO, Candesartan and Salirasib at the indicated concentrations. Cells were collected at 24 h and 48 h time points and the AnnexinV staining was performed according to the instructions from the manual (Thermo Fisher, Cat # 00-0055-56). Flow cytometry with AnnexinV-FITC conjugated antibody (Thermo Fisher, Cat # A13199) was used to determine the population of cells incorporating Annexin V and PI was used for the DNA content.

### Western Blot

Cells were lysed using Qproteome lysis buffer (Qiagen, Cat # 37901), then spun at 14,000 rpm for 10 min at 4 °C. Protein concentration in the supernatant was quantified using BCA assay (Thermo Scientific, Cat # 23227). 30 μg of protein was loaded into each lane of a TGX-stain-free pre-cast 4–20% SDS gel (Biorad, Cat # 4568094), total protein was quantified upon stain-free gel imaging, and protein was transferred onto PVDF membrane using the Trans-Blot Turbo Transfer System (BioRad, Cat # 1704150). Membranes were blocked with 5% milk in 1% TBST for 1 h, and a 1:1000 dilution of Anti-PRL-3 antibody (R&D Biosystems, Cat # MAB3219), 1:5000 phospho-ERK (Cell Signaling, Cat # 19101) or 1:5000 total ERK (Cell Signaling, Cat # 137F5) was added overnight. Following three washes in TBST, secondary HRP-conjugated antibody (GE, Cat # NA9340V) were added at a 1:5000 dilution for 1 h and membranes imaged using Clarity Western ECL Substrate. (BioRad, Cat # 1705061).

### Molecular docking

The molecular docking software, Autodock41-3 (version 4.2.6) was used to identify probable binding sites. Protein structures were obtained from published NMR solution structures of apo (PDB code: 1V3A4) and vanadate-bound (PDB code: 2MBC5, model 1) PRL-3. Ligand sdf files (pubchem.com6) were converted to PDB using OpenBabel7.8. Ligand and receptor files were prepared and Gasteiger charges added using AutodockTools1. Blind Autodock docking was performed by covering the entire protein structure with a grid box consisting of 126 × 126 × 126 points and centered at the center of the macromolecule. Docking was then performed without bias to search for all possible binding sites using Lamarckian Genetic Algorithm with a rigid protein and flexible ligands. Population size was set to 300 and the maximum number of energy evaluations and maximum number of generations were set to 30,000,000 and 27,000, respectively. For each ligand, 100 dockings were performed and clustered using an RMSD tolerance of 2 Å. In addition to the set of FDA-approved drugs, dexamethasone and PRL inhibitor I, which were used as negative and positive controls in experimental work, respectively, as well as a farnesyl tail were docked following the same protocols. Visualizations were performed using AutodockTools and PyMol10.

### Statistics

All experiments, expect for the drug screen, were performed in biological triplicate across at least three independent time points with at least two technical replicates. Where applicable, experimental values were normalized to vehicle control, ± standard deviation. Statistical tests were run in Graphpad Prism Version 9. *p* values were calculated either using one-way ANOVA with Tukey HSD or two-way ANOVA with Dunnett test for multiple comparisons, as noted in the figure legends. Changes were considered significant if *p* < 0.05.

## Supplementary Information

Supplementary Information 1.

Supplementary Information 2.

## Data Availability

All data generated or analyzed during this study are included in this article and its supplementary files. The datasets that were analyzed are available from the corresponding authors on reasonable request.
