# Supplementary material for: A screen of FDA-approved drugs identifies inhibitors of protein tyrosine phosphatase 4A3 (PTP4A3 or PRL-3)
Source: Sci Rep. 2021 May 13;11:10302. doi: 10.1038/s41598-021-89668-5 (PMC8119466; doi:10.1038/s41598-021-89668-5)
Supplement: Supplementary file 1 — Supplementary Information 1. [file 41598_2021_89668_MOESM1_ESM.pdf]

Supplemental Material

A Screen of FDA-approved drugs identifies inhibitors of Protein Tyrosine Phosphatase 4A3 (PTP4A3 or PRL-3)

Dylan R. Rivas[1#], Mark Vincent C. Dela Cerna[2#], Caroline N. Smith[1], Shilpa Sampathi[1], Blaine G. Patty[1], Donghan Lee[2,3*], Jessica S. Blackburn[1,4*]

[1.] University of Kentucky, Department of Molecular and Cellular Biochemistry, Lexington KY 40536, USA

[2.] University of Louisville, Department of Biochemistry and Molecular Genetics, Louisville, KY 20202, USA

[3.] James Graham Brown Cancer Center, Department of Medicine, Louisville KY 40202, USA

[4.] Markey Cancer Center, Lexington KY 40536, USA

*donghan.lee@louisville.edu

*jsblackburn@uky.edu

[#] These authors contributed equally to this work

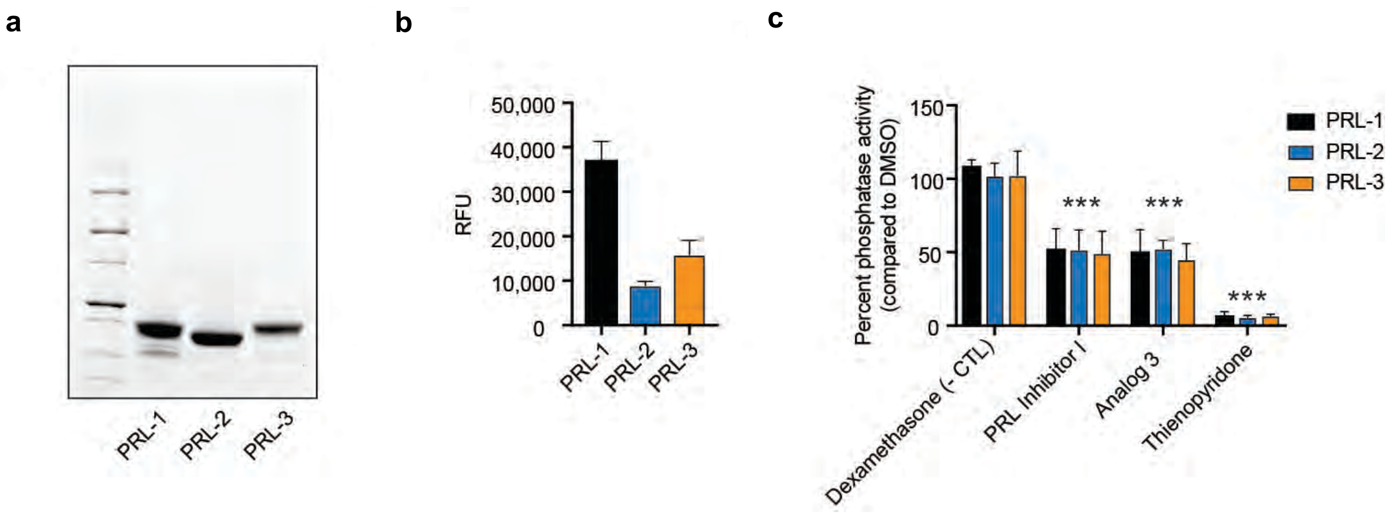

Supplemental Figure 1. Establishing the efficacy of the DiFMUP assay in assessing PRL phosphatase activity. A) SDS-PAGE gel demonstrating purity of PRL proteins used in the DiFMUP assay. B) PRL dephosphorylation of DiFMUP measured at ex=360nm and em=460nm. RFU=relative fluorescence units. C) Inhibition of PRL phosphatase activity by known inhibitors, ***p<.0001 compared to the negative control dexamethasone, an FDA-approved drug that is not expected to inhibit PRL activity. Bars represent the average of n=3, and are representative of 3 experiments. Standard deviation is shown.

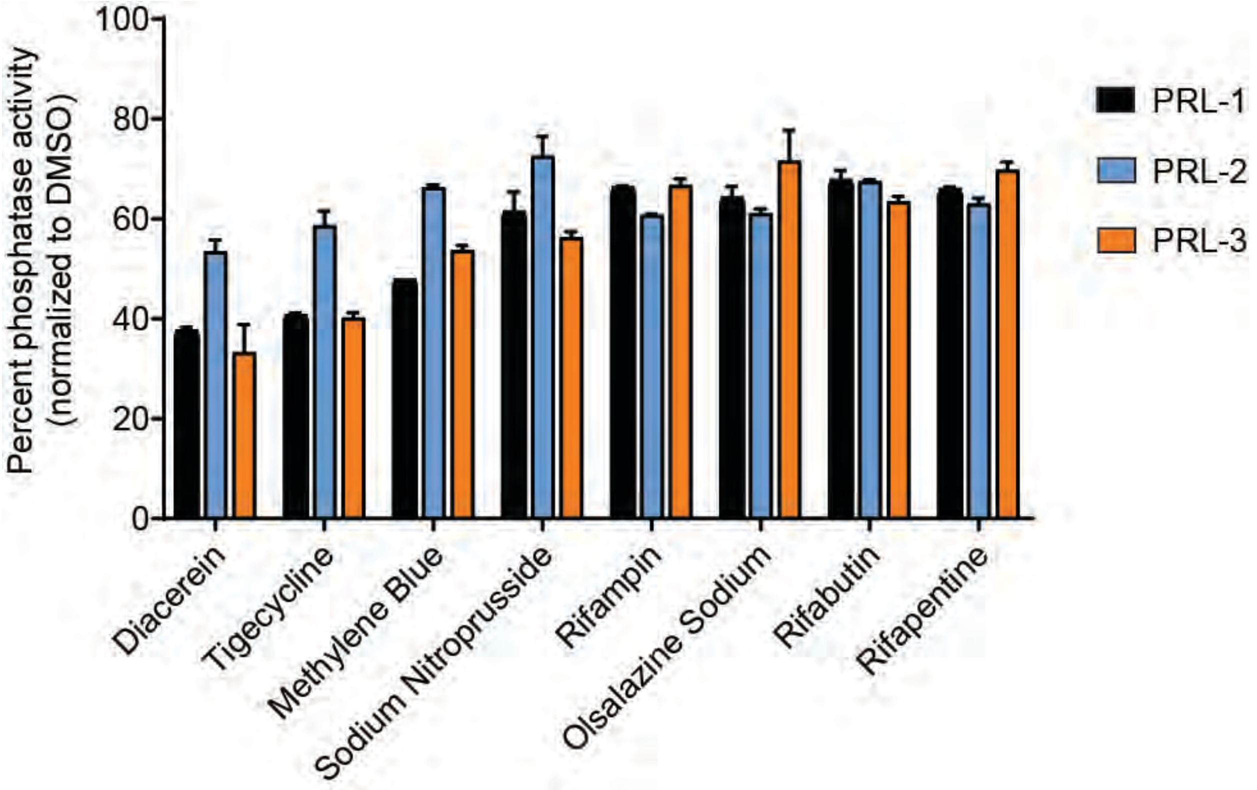

Supplemental Figure 2. A subset of hits from the initial screen failed re-analysis. The effects of broad PRL inhibitors on PRL phosphatase activity were examined. While these drugs hit in the initial screen, they did not meet the cut-off of >80% inhibition. Drugs were used at 40μM, bars are average phosphatase activity, the standard deviation is shown.

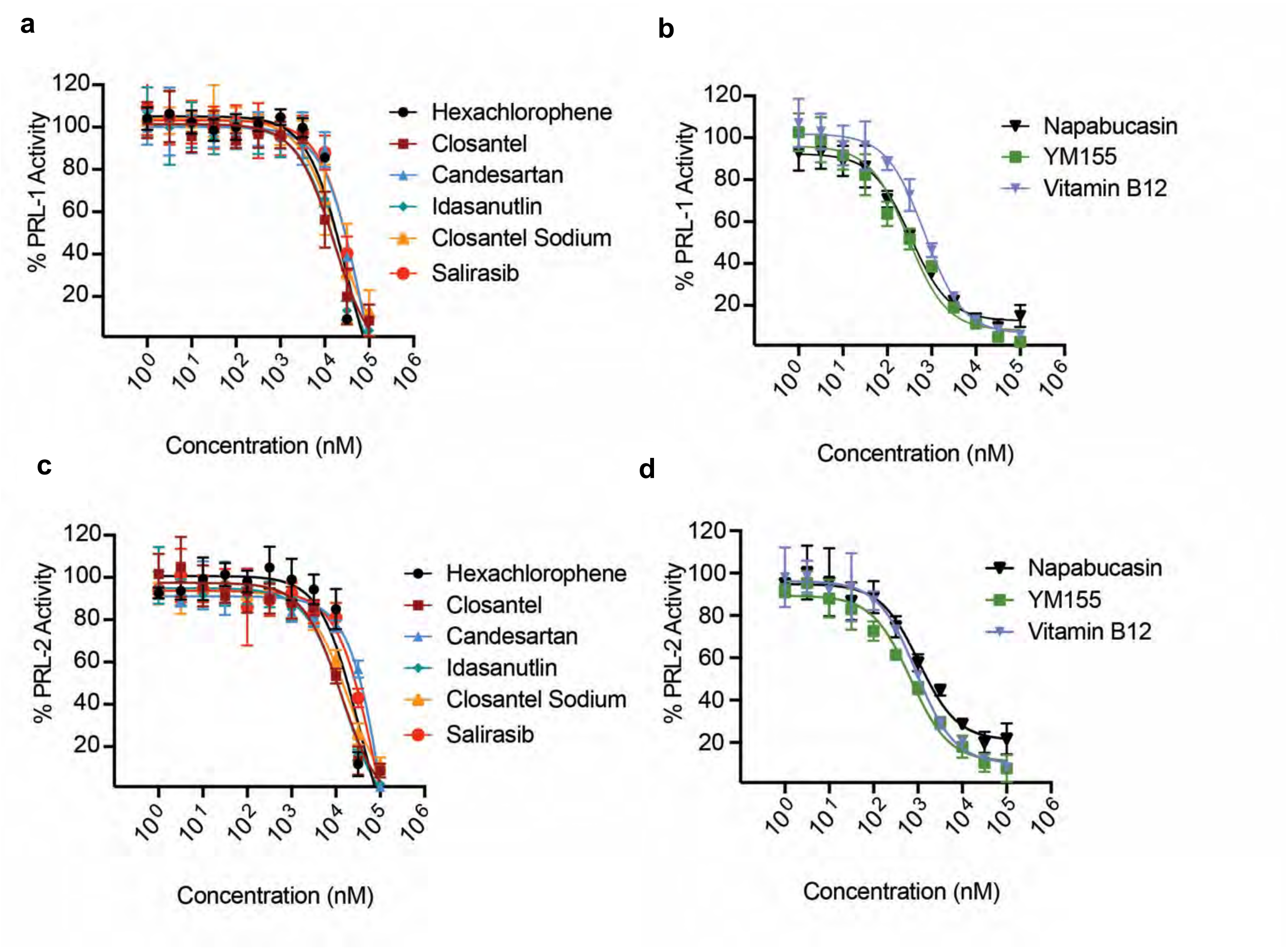

Supplemental Figure 3. Dose response curves of FDA-approved drugs against PRL-1 and PRL-2. Dose response curves showing DiFMUP phosphatase activity of PRL-1 (**a,b**) and PRL-2 (**c,d**) when treated with doses ranging between 1 nM and 100 µM of drug. Inhibition of PRL phosphatase activity by the broad PRL family inhibitors are shown, and drugs are subdivided into those with (**a,c**) high IC_{50} values and (**b,d**) low IC_{50} values

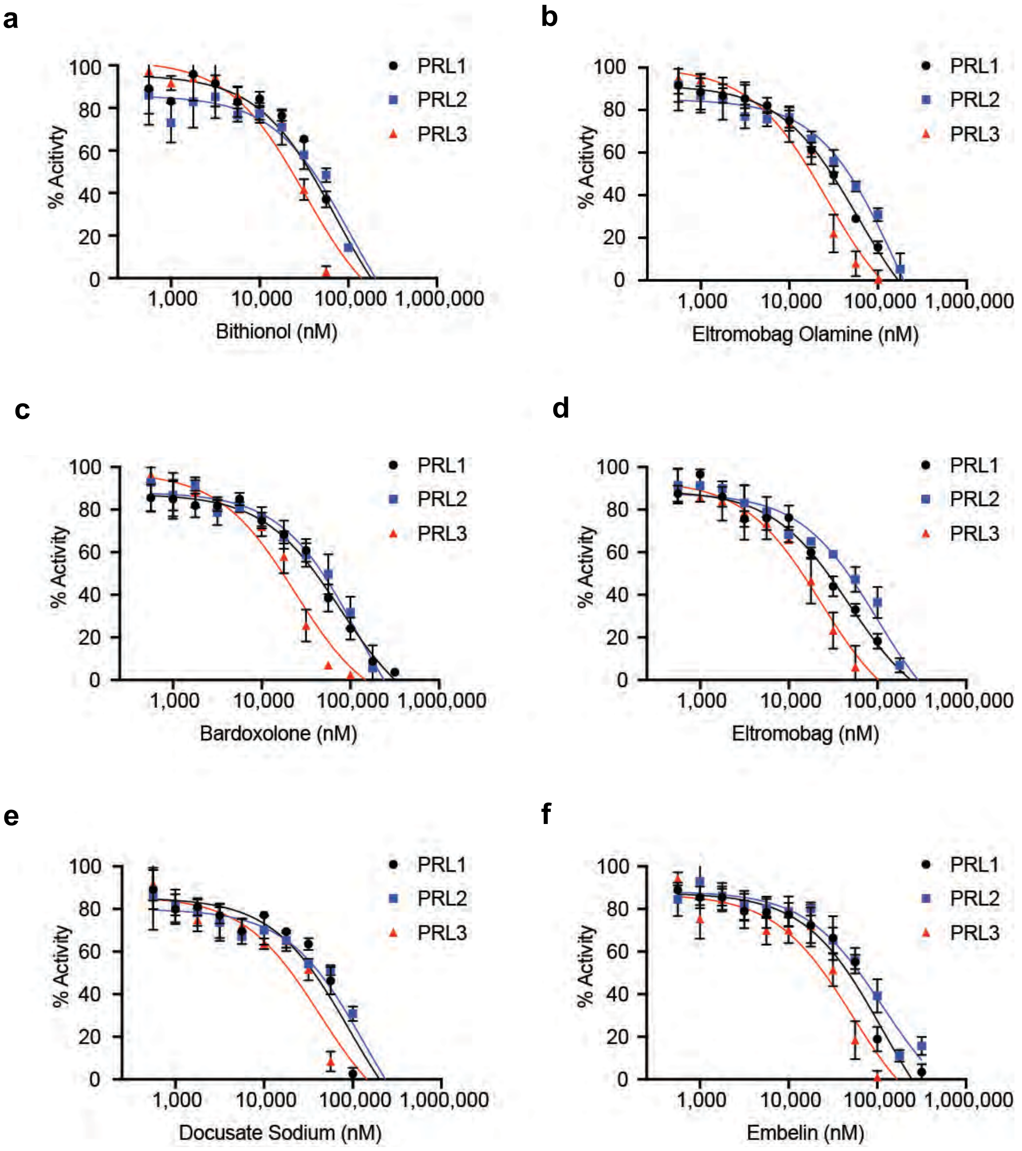

Supplemental Figure 4. Dose response curves of PRL-3 specific drugs. (a-f) Inhibition of phosphatase activity by PRL-3 specific compounds. Each data point is the average DiFMUP phosphatase activity of each PRL when treated with the drug dose noted. Assay were run in technical duplicates and are representative of three independent experiments. Standard deviation is shown.

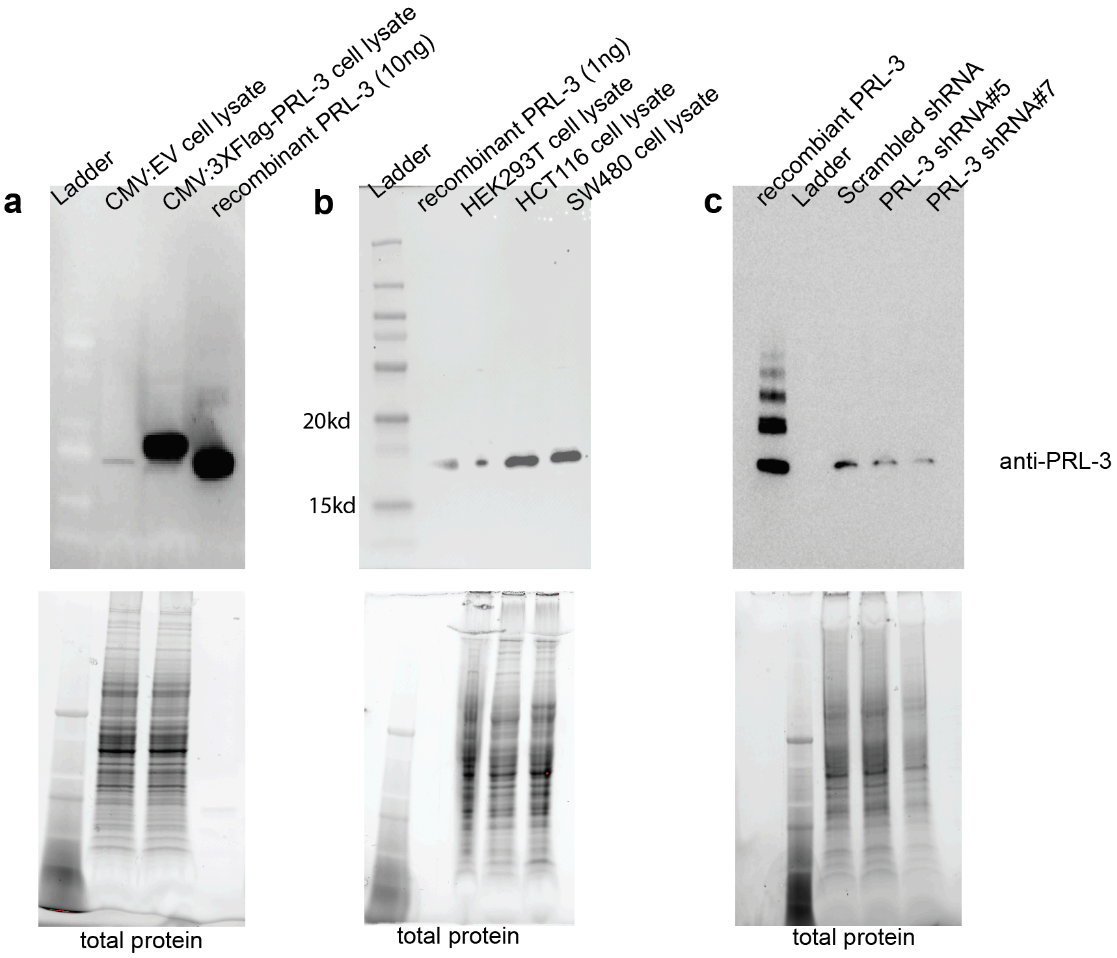

Supplemental Figure 5. PRL-3 expression in various cell lines. PRL-3 expression was assessed by western blots of cell lysates of (a) HEK293T cells transfected with the overexpression plasmids indicated, (b) HEK293T, HCT-116 and SW480 colorectal cancer cells for endogenous PRL-3, and (c) HCT-116 transfected with the shRNA plasmids indicated. Recombinant PRl-3 was used as a size control, and the total protein loaded into the gel is shown. The expected size of PRL-3 is ~18kD, with 3XFLAG-PRL-3 being slightly larger.

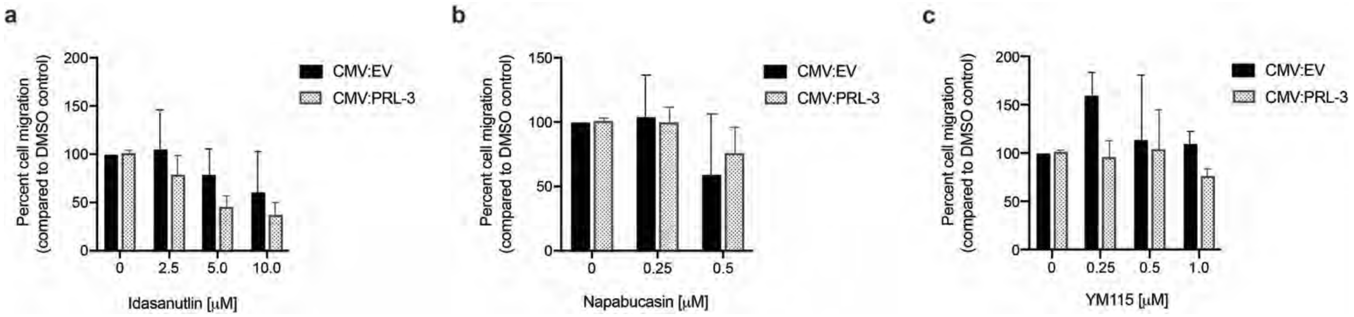

Supplemental Figure 6. Negative hits in the cell migration assay. Migration of vector control and PRL-3 overexpressing HEK293T cells after treatment with (**a**) Idasanutlin, (**b**) Napabucasin, and (**c**) YM155 at the concentrations indicated. Migration was measured as the area units migrated 24 hours after scratch. Percent migration was measured as the area units migrated 24 hours after scratch normalized to the area units migrated by 1% DMSO control treated cells withing the same dataset. All assays were run in duplicate wells, in 3 independent experiments. Statistics were done using one way ANOVA with Tukey HSD; there is no significant difference between any of the treatment groups.

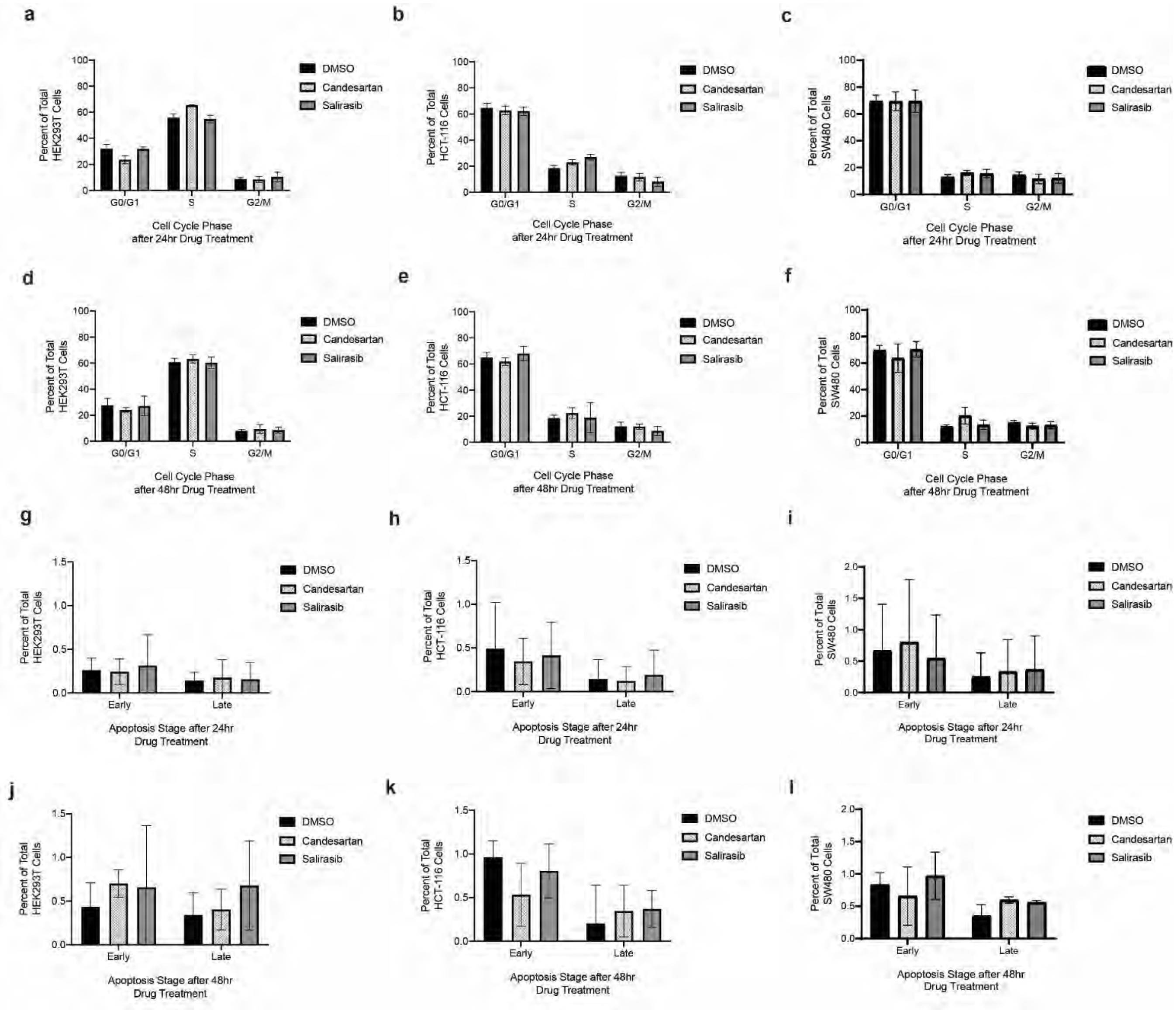

Supplemental Figure 7. Salirasib and Candesartan did not significantly impact cell cycle or apoptosis in HEK293T, HCT-116 or SW480 cells. Flow cytometry analysis of EdU staining to assess cell cycle in HEK293T (**a**), HCT-116 (**b**), and SW480 (**c**) cells treated with either 1% DMSO, 100 μM Salirasib or 25 μM Candesartan for 24hr. (**d-e**) shows cell cycle effects after 48hr drug treatment, in the same cell lines. Apoptosis of cells treated with either 1% DMSO, 100 μM Salirasib or 25 μM Candesartan for 24hr was quantified by AnnexinV plus propidium iodide staining. Flow cytometry was used to quantify staining in HEK293T (**g**), HCT-116 (**h**), and SW480 (**i**) cells. (j-l) shows AnnexinV + propidium iodide staining on the same cell lines treated with drug for 48hr. There is no significant difference in any of the groups, compared to DMSO, using a two-way ANOVA with Dunnett's correction.

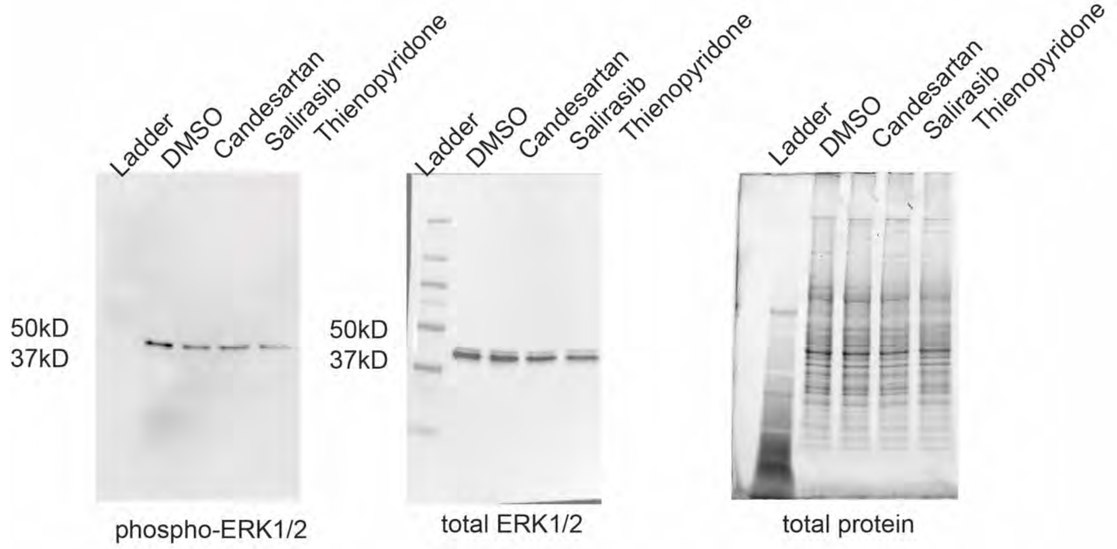

Supplemental Figure 8. Phospho-ERK1/2 is reduced in drug treated cells. HCT116 colorectal cancer cells were treated for 24 hr with either 1% DMSO, 100 µM Salirasib, 25 µM Candesartan, or 25 µM Thienopyridone, a known PRL inhibitor. PRL-3 has been previously found to regulate ERK signaling in colorectal cancer cells. Expected size of ERK1/2 is 44kD and 42kD, respectively. Total protein is shown.

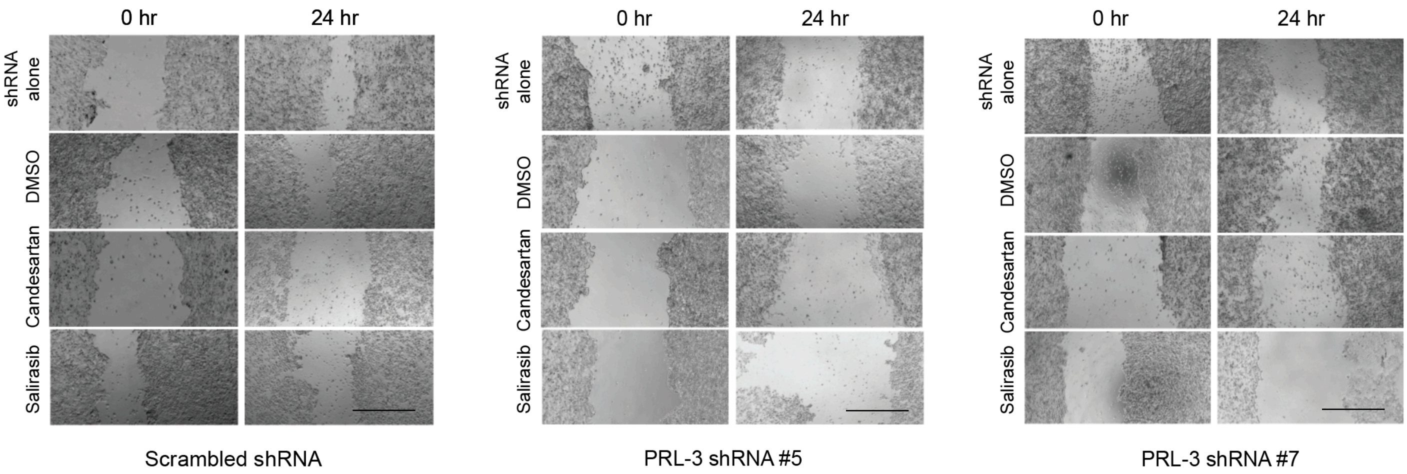

Supplemental Figure 9. Representative images from scratch assays presented in Figure 5. HCT-116 cells were transfected with expression plasmids containing either scrambled/non-targeting shRNA, or shRNAs targeting PRL-3 (#5 and #7). Cells were selected with puromycin for 48 hours then plated as a monolayer of cells. The monolayer was scratched with a pipet tip and immediately imaged (0 hr) and imaged again at 24 hr post-scratch. In some experiments, at the 0 hr timepoint, cells transfected with shRNA were additionally treated with 1% DMSO, 25 µM Candesartan, or 100 µM Salirasib. For all, scale bar = 1mm.

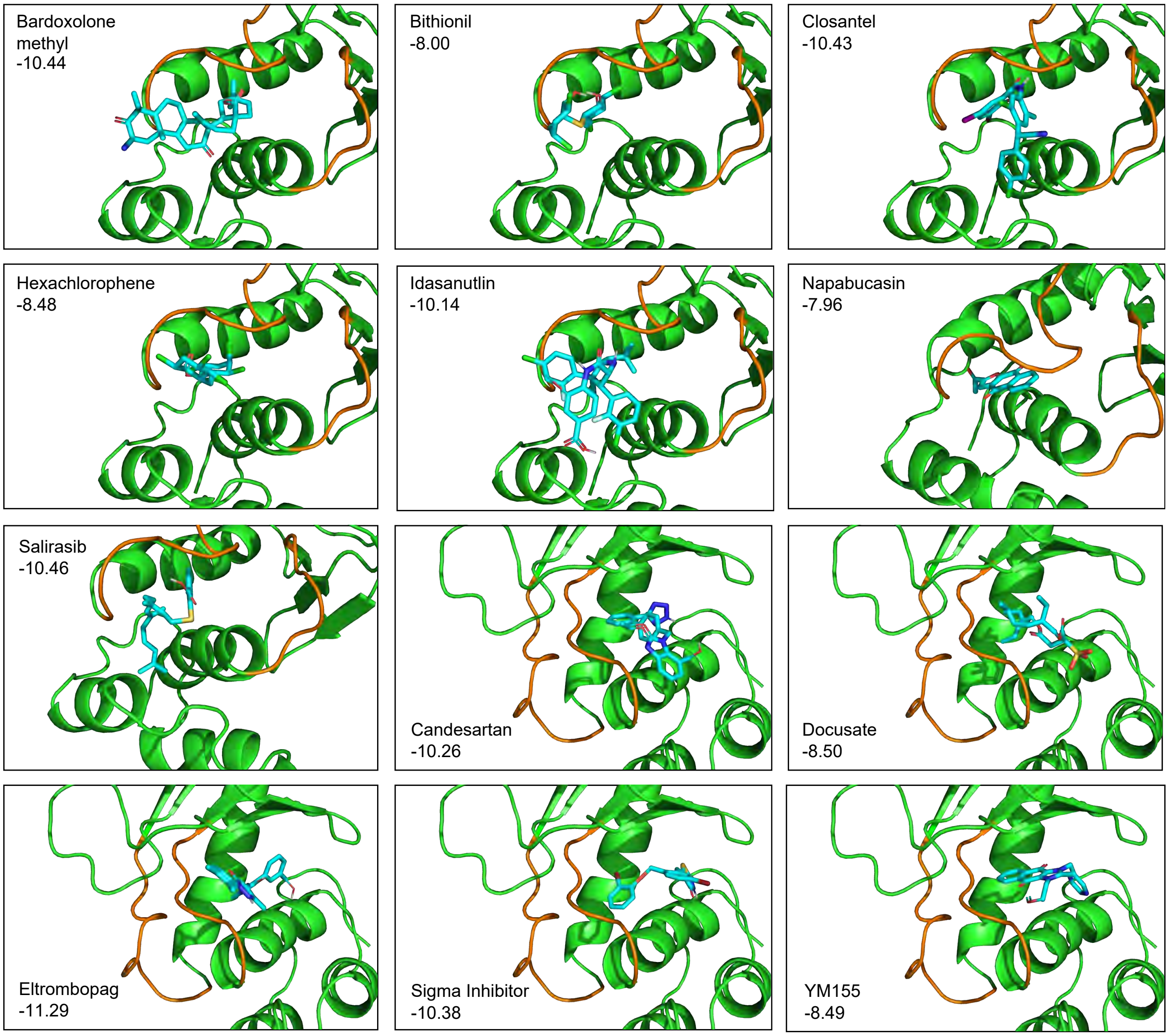

Supplemental Figure 10. FDA-approved drugs docked onto allosteric sites of PRL-3 in closed conformation. The binding energy for each drug is shown.

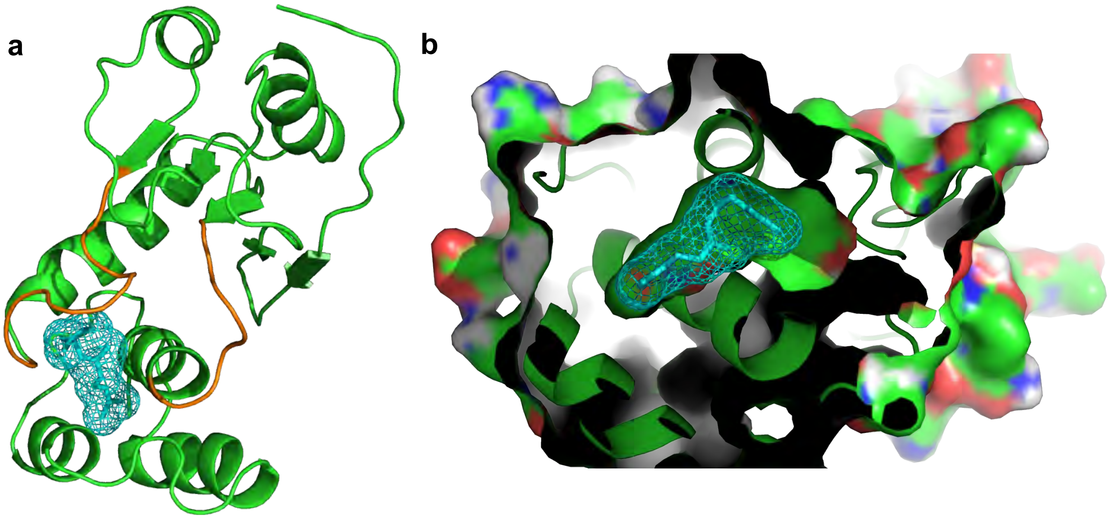

Supplemental Figure 11. Blind docking identifies Site 1 as a potential binding site for a farnesyl tail.

Supplemental Table 1, File name: Supplemental table 1_screen.xlsx

A Screen of FDA-approved drugs identifies inhibitors of Protein Tyrosine Phosphatase 4A3 (PTP4A3 or PRL-3)

Dylan R. Rivas[1#], Mark Vincent C. Dela Cerna[2#], Caroline N. Smith[1], Shilpa Sampathi[1], Blaine G. Patty[1], Donghan Lee[2,3*], Jessica S. Blackburn[1,4*]

1. University of Kentucky, Department of Molecular and Cellular Biochemistry, Lexington KY 40536, USA

2. University of Louisville, Department of Biochemistry and Molecular Genetics, Louisville, KY 20202, USA

3. James Graham Brown Cancer Center, Department of Medicine, Louisville KY 40202, USA

4. Markey Cancer Center, Lexington KY 40536, USA

*donghan.lee@louisville.edu

*jsblackburn@uky.edu

[#] These authors contributed equally to this work

Supplemental Table 2: Candesartan and Salirasib have no negative effect on activity of other phosphatases (at 25µM)

Phosphatase Tested	Percent Phosphatase Activity (compared to DMSO)	
	Candesartan	Salirasib
ACP1 (LMPTP-A)	97	96
ACP1 (LMPTP-B)	113	106
CD45	100	102
Lambda PP	107	112
MKP5	99	106
PP1alpha	105	104
PP2A	110	107
PTP1B	109	114
PTPbeta	85	91
PTPMEG	109	107
PTPMEG2	97	102
PTPN22	136	129
PTPRM	107	111
SHP1	116	113
SHP2	104	103
TCPTP	128	118
VHR	87	97
Yoph	112	102

Supplemental Table 3:
Docking results of FDA-approved drugs with the phosphatase PRL-3 in the open and closed forms.

Closed Conformation (PDB: 2MBC)	Est. Free Energy of Binding[a]	Est. K_i (nM)[b]	Intermolecular Energy	Total Internal Energy	Torsional Free Energy	Unbound System Energy	Site[c]
Bardoxolone-Me	-10.44	22	-11.25	-0.23	+0.60	-0.44	1
Bithionil	-8.00	1370	-7.76	-1.98	+1.19	-0.54	1
Candesartan	-10.26	30	-12.54	-1.83	+2.39	-1.73	2
Closantel	-10.43	23	-11.42	-2.06	+1.19	-1.86	1
Docusate	-8.50	586	-13.00	-1.36	+5.37	-0.49	2
Eltrombopag	-11.29	5	-12.52	-1.81	+2.09	-0.96	2
Hexachlorophene	-8.48	605	-8.46	-1.92	+1.19	-0.71	1
Idasanutlin	-10.14	37	-10.79	-5.32	+2.68	-3.29	1
Napabucasin	-7.96	1470	-8.26	-0.13	+0.30	-0.13	1
Salirasib	-10.46	22	-12.86	-1.53	+3.28	-0.65	1
YM155	-8.99	257	-9.99	-0.86	+1.49	-0.36	2
Dexamethasone	-8.49	596	-8.68	-1.58	+1.49	-0.28	1
Sigma Inhibitor	-10.38	25	-11.56	-0.86	+1.19	-0.84	2
**Open Conformation (PDB: 1V3A)**	**Est. Free Energy of Binding**	**Est. K_i (nM)**	**Intermolecular Energy**	**Total Internal Energy**	**Torsional Free Energy**	**Unbound System Energy**	**Site**
Bardoxolone-Me	-8.54	553	-8.90	-0.67	+0.60	-0.44	In
Bithionil	-7.74	2100	-8.82	-0.65	+1.19	-0.54	In
Candesartan	-8.51	577	-9.36	-3.27	+2.39	-1.73	In
Closantel	-9.00	254	-9.94	-2.1	+1.19	-1.86	In
Docusate	-8.38	726	-11.83	-2.38	+5.37	-0.46	O
Eltrombopag	-10.64	16	-11.80	-1.88	+2.09	-0.96	In
Hexachlorophene	-9.01	247	-9.02	-1.89	+1.19	-0.71	In
Idasanutlin	-9.65	84	-10.45	-5.2	+2.68	-3.31	In
Napabucasin	-6.70	12340	-6.99	-0.13	+0.30	-0.13	In
Salirasib	-9.00	253	-11.06	-1.87	+3.28	-0.65	In
YM155	-7.92	1560	-8.65	-1.12	+1.49	-0.35	O
Dexamethasone	-7.87	1690	-8.35	-1.30	+1.49	-0.28	In
Sigma Inhibitor	-9.52	105	-10.36	-1.20	+1.19	-0.84	In

[a] Estimated Free Energy = Intermolecular + Total Internal + Torsional – Unbound Energy
[b] Autodock estimates inhibition constant from the estimated free energy
[c] Site 1 and 2 refer to the allosteric sites (see text), while In and O refer to a site in between the loops, or outside of this area.

Supplemental Table 4: Compounds used in this study

Manufacturer	Compound	Catalog Number
Selleck	Hexachlorophene	S4632
Selleck	Closantel	S4106
Selleck	Candesartan Cilexetil	S2037
Selleck	Vitamin B12	S1902
Selleck	Idasanutlin	S7205
Selleck	Salirasib	S7684
Selleck	YM155	S1130
Selleck	Closantel Sodium	S4105
Selleck	Napabucasin	S7977
Selleck	Bithionol	S4552
Selleck	Eltrombag Olamine	S2229
Selleck	Eltrombag	S4502
Selleck	Bardoxolone Methyl	S8078
Selleck	Embelin	S7025
Selleck	Docusate Sodium	S4588
Sigma	PRL-3 Inhibitor I	P0108
Enamine	Analog 3	Z44389470
Enamine	Thienopyridone	EN300-171034
